# The Use of Iron as a Prophylactic against Cholera

**Published:** 1849-01

**Authors:** 


*The use of Iron as a Prophylactic against Cholera.*—I wish to suggest to those exposed to the influence of cholera, the internal use of iron as a prophylactic.

I conjecture that when the blood is well impregnated with iron, it is rendered less prone to undergo the morbid change in which many epidemic diseases primarily consist. The experience of an individual is insufficient to put this conjecture to the test; and as regards cholera, I have not even that experience to offer. During the prevalence of Irish fever, I believe I did obtain a little negative evidence in support of my opinion, but not nearly sufficient to establish it.

Taken in the form of pill along with solid food, iron scarcely ever disagrees, provided neither fever nor active inflammation be present. Any one disposed to try it against the contagion—for such I believe it—of cholera, will find a grain or two of the sulphate, made into a pill, with extract of gentian, to be taken during, or immediately after, each of the principal meals, a convenient method.—M. D.—*Lancet.*

